# Detecting Binge Drinking and Alcohol-Related Risky Behaviours from Twitter’s Users: An Exploratory Content- and Topology-Based Analysis

**DOI:** 10.3390/ijerph17051510

**Published:** 2020-02-26

**Authors:** Cristina Crocamo, Marco Viviani, Francesco Bartoli, Giuseppe Carrà, Gabriella Pasi

**Affiliations:** 1 Department of Medicine and Surgery, University of Milano-Bicocca, 20126 Milan, Italy; francesco.bartoli@unimib.it (F.B.); giuseppe.carra@unimib.it (G.C.); 2 Department of Informatics, Systems, and Communication, University of Milano-Bicocca, 20126 Milan, Italy; gabriella.pasi@unimib.it

**Keywords:** binge drinking, vulnerability, risky health behaviour, user-generated content, social media analytics, data science, supervised machine learning

## Abstract

Binge Drinking (BD) is a common risky behaviour that people hardly report to healthcare professionals, although it is not uncommon to find, instead, personal communications related to alcohol-related behaviors on social media. By following a data-driven approach focusing on User-Generated Content, we aimed to detect potential binge drinkers through the investigation of their language and shared topics. First, we gathered Twitter threads quoting BD and alcohol-related behaviours, by considering unequivocal keywords, identified by experts, from previous evidence on BD. Subsequently, a random sample of the gathered tweets was manually labelled, and two supervised learning classifiers were trained on both linguistic and metadata features, to classify tweets of genuine unique users with respect to media, bot, and commercial accounts. Based on this classification, we observed that approximately 55% of the 1 million alcohol-related collected tweets was automatically identified as belonging to non-genuine users. A third classifier was then trained on a subset of manually labelled tweets among those previously identified as belonging to genuine accounts, to automatically identify potential binge drinkers based only on linguistic features. On average, users classified as binge drinkers were quite similar to the standard genuine Twitter users in our sample. Nonetheless, the analysis of social media contents of genuine users reporting risky behaviours remains a promising source for informed preventive programs.

## 1. Introduction

Excessive alcohol use is a frequent risky behaviour, which accounts for between 1.3% and 3.3% of health costs globally [1]. High rates of alcohol consumption and heavy drinking are common among young people, raising concerns in terms of public health issues [2]. Binge drinking (BD) is defined as four or more drinks for women and five or more for men on a single occasion [3], with current rates of up to 27% both in the United States and Europe [4,5]. The use of the term is popular and clearly recognizable not only to researchers in the field but also to the general public and young people in particular [6]. Young adults who engage in BD are more likely to report other health risks such as riding with drunk drivers, smoking cigarettes, being a victim of violence, attempting suicide, or using illicit drugs [7]. In addition, knowledge and perception of BD risks are often limited [8,9] among young people, with impaired decision making playing a major role [10] in actions leading to immediate rewards, poor skills in terms of anticipating negative consequences and learning from previous mistakes, considering consequences not relevant to themselves [11,12]. IT-based evidence has shown encouraging results as regards alcohol use reduction and behavioural support among young people (e.g., [13]). This is likely to be due to young people’s propensity to use electronic devices and their expertise with them (e.g., smartphones) to engage with social media [14]. Previous research explored vulnerability to addiction and risky behaviours across big data by identifying clusters according to individuals’ personal characteristics and circumstances, and by comparing different techniques in terms of methodological reliability [15,16].

Social media platforms are increasingly popular among both young people and individuals belonging to different age groups; they combine media and peer influences from a broad range of areas involving social norms, risk perceptions, and related behaviours [17]. Indeed, social influences affect drinking behaviours, and online social networks can have an effect on both the style and the amount of drinking behaviours also in farthest circles [18,19]. Specifically, people share online information and access contents that other subjects have posted on the Web, including their own experiences, which defines a new IT user paradigm [20,21]. This scenario, completely different from an end-user condition, enables the access to social media, where large amounts of User-Generated Content (UGC) are spread every day across virtual communities, almost without any external control [22,23,24], lowering at the same time the perception of anonymity and confidentiality issues among users [25,26]. This applies particularly to those topics that people are reluctant to discuss with healthcare professionals, including behaviours, opinions, and individual-directed actions that are difficult to track and measure in a clinical setting [27].

### 1.1. Binge Drinking and Social Media

Previous evidence showed that, for instance, young people frequently discuss their drinking behaviour on social media [28], alcohol misuse contents are easily shown on users’ profiles [29,30], and exposure to drinking-related content contributes to the normalization of drinking [31]. Indeed, impulsivity features typical of BD and similar alcohol-related risky behaviours may fit particularly well into social media, whose users can easily and instantly connect to a mass audience via brief messages [32]. Furthermore, a recent systematic review and meta-analysis drew actually attention to a moderate strength of relationship between exposure to alcohol-related social media content and alcohol consumption and consequences, with study participants frequently discussing their drinking behaviour on social networking sites [28]. Risky behaviour- and substance-related research has used Twitter databases to the aim of mining data to explore sentiment, topics and sources for Tweets related to tobacco [33], marijuana [34], alcohol [35] or a combination of different substances [36]. In [33], the authors explored tweets to learn more about the use of tobacco by adopting an unsupervised clustering algorithm to group tweets. Further studies investigated the categorization of substance related content (i.e., cannabis and synthetic cannabinoid) by using supervised machine learning with fairly high accuracy [37], and by tracking changes in users’ opinions in Twitter over time and across different regions. Interestingly, recent evidence demonstrated the popularity of drinking-related chatter in particular on Twitter, with most alcohol-related tweets reflecting a positive sentiment toward alcohol use, outnumbering anti-alcohol Tweets, and with references to heavy drinking behaviours [35]. Tweets normalizing or encouraging marijuana use over alcohol use are reported to be even more common [36].

### 1.2. The Current Study

In order to explore features that are hardly detectable by using classical epidemiological designs, an alternative, yet consistent, approach may involve BD-related UGC, by employing a data-driven process to investigate public health concerns at a reduced cost [27,38]. The available tools for automatic content classification may be fruitfully employed to analyse tweets related to alcohol and drug recreational use, to the aim of harnessing social media platforms for alcohol and drug misuse surveillance research [37]. In particular, the study reported in this paper was aimed to explore the communication of risky behaviours on Twitter, grounded on the hypothesis that there might be a relationship between alcohol-related shared information as available from social media posts and risky behaviours such as BD. A better understanding of how people are involved in social networks about alcohol-related behaviours could help in finding innovative ways of promoting healthy behaviours and in establishing potential preventive programmes. Therefore, we aimed at mapping clusters of tweets that explore a semantic spectrum of alcohol-related UGC, by identifying both the language and the common topics discussed by potential binge drinkers.

## 2. Materials and Methods

**Figure 1:**
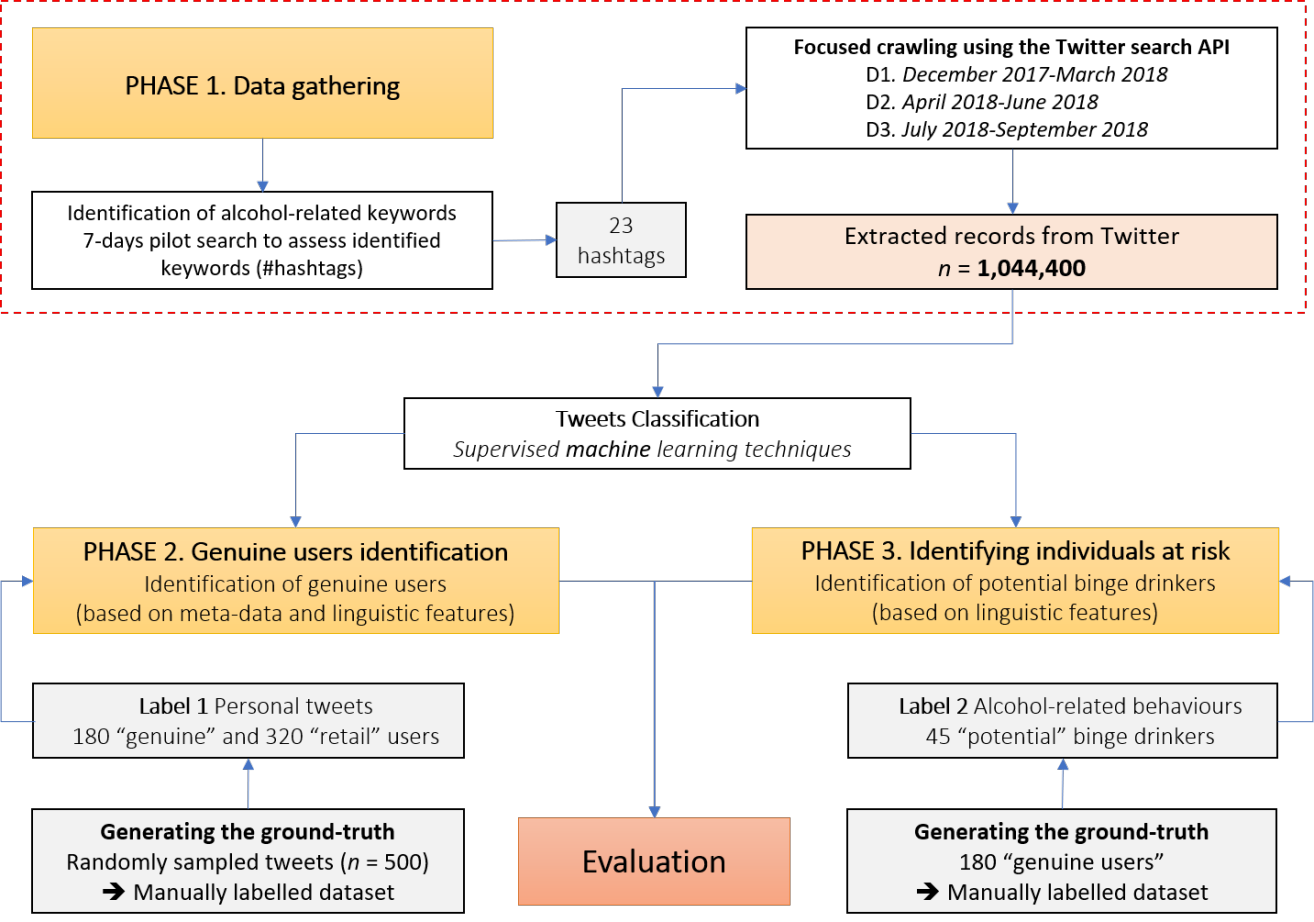
Workflow used to characterize alcohol-related risky behaviours in Twitter.

We considered the Twitter social media platform, a free-to-use microblogging site, characterized by immediacy and easiness of use [39], with over 200 million users internationally [40]. Twitter involves unidentified people, who can instantly connect to a mass audience via brief messages (280 characters or less, i.e., “tweets”), displayed on both the author’s homepage and those of his/her followers [41]. It represents an ideal public place to hear the latest news, exchange ideas and connect with people, in real time with an impressive volume of around 500 million tweets per day [42], producing a considerable amount of unstructured data. Thus, Twitter can be considered as a key source of social media contents, since it provides feasible access to data (via Advanced Programming Interfaces—APIs and suitable libraries) both retrospectively on sets of historical tweets connected to specific users, and prospectively to capture several matching tweets and related metadata [43], thus allowing to study also individuals’ health behaviours, such as drug and alcohol use [27]. To develop a specific approach for our condition of interest, i.e. BD, a structured workflow was followed, with three distinct phases (Figure 1).

In Phase 1, a dataset was built by gathering alcohol-related tweets and related metadata from the microblogging platform, by focusing on BD-related hashtags identified by a panel of experts. Phase 2 dealt with the automatic identification via supervised classification of genuine unique users (intended as real persons) with respect to the Twitter accounts of media and business activities, and social bots, by considering different characteristics connected to both tweets’ content and metadata. Finally, in Phase 3, supervised classification was applied to automatically identify potential binge drinkers among genuine users, focusing only on linguistic features.

### 2.1. Phase 1: Data Gathering

Eligible records were Twitter posts (i.e., tweets) quoting alcohol-related behaviours. In particular, only tweets written in English and containing specific keywords addressing the condition of interest were gathered. Relevant keywords were constituted by some hashtags identified according to previous evidence on BD and by exploring the platform through a systematic search by a panel of experts. Hashtags are labels preceded by the # symbol (technically metadata tags), and they are generated by users on Twitter to allow an easy identification of a specific topic on a dynamic thread of tweets. The search phase for BD-related hashtags from Twitter was carried out for one week; they belong to distinct categories:

- Hashtags concerning alcoholic beverages, e.g., #alcohol, #cocktail, #drinks, #rum;
- hashtags that indicate phenomena known to be typical scenarios of excessive alcohol use, such as “pub crawling” which indicates the action of drinking in different pubs on the same evening, e.g., #pubcrawl, #pubcrawling, #botellon;
- hashtags that explicitly indicate the common after-effects of drinking too much alcohol, or the condition of drunkenness, e.g., #wasted, #hangover, #toomuchalcohol, #sorehead, #drunkies, #drunkasfuck;
- hashtags that contain a direct reference to binge drinking, e.g., #bingedrinking.

The complete list of the 23 hashtags used to filter tweets related to alcohol consumption is as follows: #alcohol, #alcoholic, #alcoholics, #bingedrinking, #botellon, #cocktail, #cocktails, #drinking, #drinks, #drunk, #drunkasfuck, #drunkennights, #drunkies, #getdrunk, #hangover, #nomorealcohol, #pubcrawl, #pubcrawling, #rhum, #sorehead, #toomuchalcohol, #vodka, #wasted. Based on the selected hashtags, a systematic focused crawling process [44] (i.e., focused on the specific hashtags) through public APIs was implemented on Twitter, taking into account Twitter updates in terms of tweets length (currently 280 characters vs. 140 before) using an ad-hoc Python script [45]. During the crawling process, specific available data about tweets and their authors were recorded. These data included:

(*i*) The entire text of the tweet, discarding multimedia content;
(*ii*) metadata associated with the tweet, such as the reactions to the tweet, expressed via retweets and likes, the date and time the tweet was created, information on geo-location if available;
(*iii*) details of the original tweet if the post was a retweet (including information about the original author of the tweet);
(*iv*) author’s details such as screenname (also known as handle), complete name, biography, number of tweets in their timeline, number of followees and followers, date of account creation.

Twitter data were gathered with respect to three different time periods, i.e., from December 2017 to March 2018, from April 2018 to June 2018, and from July 2018 to September 2018. These represent approximately three seasonal intervals, i.e., winter, springtime, and summer. These tweets were therefore split into three datasets: D1, D2, and D3. Based on the gathered tweets, we were able to collect also the thread of tweets related to single users of interest, thus constituting a new dataset D4.

### 2.2. Phase 2: Identification of Genuine Users with Respect to Bots, Media, and Business Accounts

To ensure that collected tweets were suitable for the analytical algorithms, a source classification aimed at removing “source noise” [37] was carried out before the identification of potential binge drinkers. Specifically, in this phase, we separated tweets belonging to personal accounts from bot-, media- and business-related tweets, since these sources generated inappropriate contents. These included educational messages and videos for problems related to alcohol; news reports or blogs with food-related content; businesses accounts and bartenders advertising alcohol premises.

#### 2.2.1. Supervised Learning for the Identification of Genuine Users

Several approaches have been proposed in the literature to distinguish user-generated tweets (personal communications) from spam components or automated programs, assigning “objects” characterized by specific features to two (or more) predefined classes [46,47,48]. This task is generally accomplished by means of supervised machine learning techniques by using (i) a subset of the objects already labelled with respect to the class to which they belong, and (ii) a vector of characteristics (i.e., features), associated with the objects to be classified. In supervised learning, a model (e.g., a classification model) is trained on the labelled objects (i.e., the training set) by considering the values of the features associated with the labelled objects; then, the resulting model can be applied to unlabelled objects (i.e., the test set) to automatically assign them a class.

To this purpose, in this work, a binary classification algorithm was applied to a training set containing both tweets written by genuine users and tweets written by bots or media/business, which were manually labelled. In order to build a labelled set of tweets written by genuine users we randomly selected a subsample of tweets from the gathered datasets by considering around 500 distinct users (either genuine or non-genuine) and their associated tweets (each user had on average two tweets in the gathered datasets). Based on a manual analysis of those users, we were able to label 320 users as non-genuine, while 180 users were identified as to be likely genuine users. The feature vectors identified to classify personal tweets with respect to bot-, media-, and business-related tweets (and, hence, real users with respect to non-real ones), included the following features:

1. The number of tweets of a single account: Users with a high number of tweets are probably media, commercial, or bot accounts [46];
2. the average number of hashtags per tweet: Hashtags are the “keywords” by which users identify the main topics contained in their message. A genuine user is expected to include a limited number of hashtags in a single tweet, while those who want to promote their own content often abuse of hashtags to increase the probability to find their content when using search engines [48,49];
3. the average number of mentions per tweet: Mentions, i.e., citing another Twitter account by the use of the symbol ‘@’ followed by the name of another user, for conversation and discussion purposes. These interactions are more specific of real people, while commercial activities often send general messages and do not hold individual conversations with their circle of followers [49];
4. the number of occurrences of personal pronouns per tweet: The use of personal pronouns is strictly connected to people. Advertising messages are often written in a “dry” and impersonal form [49];
5. the average number of URLs per tweet: Links to external sites (often more than one) are frequently posted by commercial activities to move users’ browsing from Twitter to their brand’s site [46];
6. the presence of URLs in the user profile: Commercial activities extensively use the platform’s advertising potential;
7. the retweet/tweet ratio: Genuine users rarely re-tweet without comments, whereas accounts retweeting about a brand behave in RSS feed style [48];
8. the network size: Profiles with a large number of followees and followers are likely to represent a famous person or a company;
9. the followers/followees ratio: For genuine user accounts, this ratio does not deviate too far from the unit. It is reasonable to expect that one person follows a certain number of profiles in a reciprocal way. Often the imbalance is severe for famous people and businesses that tend to have a high number of followers (even in the order of tens or hundreds of thousands of units) but very few or even zero followees (because the purpose of that account is not to read the contents published by third parties);
10. the presence of geo-located tweets: The use of Twitter occurs mainly via its mobile app, often with geo-localization turned on; on the other hand, desktop use is typical of business users [50];
11. the number of “bad tokens” per tweet: Along with the features described above, we identified by manual inspection of a random sample of some users’ tweets, some words (bad tokens) that likely indicate a non-personal profile. Since a high number of occurrences of bad tokens suggests that the tweet has been written by a business or a bot, they were automatically eliminated from the dataset by using a Python script through the Natural Language Toolkit framework (NLTK) [51].

#### 2.2.2. Classifying Genuine Users

Based on the selected features, and by employing the training set composed of the 500 users mentioned in the previous section, two classifiers were trained, tested and evaluated, based on two well-known supervised models: Support Vector Machines (SVMs) and Random Forests (RFs) [46,48,49].

##### Normalization

Due to the different ranges of values associated with the features, these were normalized in a common range before being analyzed by the (supervised learning) classifiers. For example, the average number of URLs per tweet is a real number, while the presence of the URL in the user profile is a value that can assume values only in the set {0,1}. To obtain a common scale, each value $x_{i}$ associated with feature *i* has been normalized according to the following formula:

(1) $$x_{i}^{\prime }=\frac{x_{i}-\mu_{i}}{\sigma_{i}},$$

where $x_{i}^{\prime }$ is the value $x_{i}$ normalized in the [0,1] interval, $\mu_{i}$ and $\sigma_{i}$ are the mean and the standard deviation of feature *i* with respect to the associated values referred to the training data. Formally,

(2) $$\mu_{i}=\frac{1}{N}\sum_{j=1}^{N}x_{ij}$$

and

(3) $$\sigma_{i}=\sqrt{\frac{1}{N}\sum_{j=1}^{N}{\left(x_{ij}-\mu_{i}\right)}^{2}},$$

where *N* is the cardinality of the training set.

##### Cross-Validation

In addition, we had to handle the relatively limited size of the training data, since the labelled tweets associated with the users’ profiles represented a subset of the entire dataset of tweets gathered during Phase 1. This limitation was unavoidable given the manual labelling of the considered datasets, which were composed of around a million of tweets. Cross-validation is a well-known technique that may be used to evaluate the results of a model (i.e., a classifier). It enables the use of a limited sample of labelled data in order to estimate how the model is expected to perform when used to make predictions on data not considered during the model training. Specifically, we performed a *k*-fold cross-validation, with a value of *k* equal to 5. By this strategy, the available labelled dataset was split into five subsets, and the classifier was trained and evaluated five times. At each of the five training and evaluation rounds, 4 (i.e., 5-1) subsets were used to train the model, and the remaining subset was used as a test set to validate the model. The test set changes sequentially at each round. Evaluation results from the five evaluation rounds have been then summarized to obtain a final estimate of effectiveness for the considered classifier.

The framework used for the implementation of the selected classifiers (i.e., SVM and RF) was the scikit-learn (sklearn) library, which constitutes a usual choice for machine learning applications in Python [52]. In particular, for both the normalization and cross-validation processes, the methods of preprocessing.StandardScaler and model_selection.cross_validate classes of sklearn were used. The two classifiers have been evaluated through Receiver Operating Characteristic (ROC) curve analyses with Area Under the resulting ROC Curve (AUC) showing the discrimination capability of classifiers at different operating points.

### 2.3. Phase 3: Identifying Potential Binge Drinkers

After distinguishing genuine from non-genuine users, we implemented an additional classifier focusing only on linguistic features (i.e., features connected only to the text of the tweets), since these may allow to capture similarities in the vocabularies used by potential binge drinkers and, possibly, automatically identify them. To extract linguistic features we considered, for each user, her/his set of tweets as a unique text, i.e., a document. Technically speaking, through the CountVectorizer class of the sklearn framework, we counted for each word its number of occurrences in a document. We then computed for each word and each document (through the TfidfTransformer class) the so called tf-idf value, a weight that combines the number of occurrences of a word in a document with the frequency of the word in the whole collection. The tf-idf values associated with each word in each user’s tweet constitute feature values considered to the classification purpose.

**Figure 2:**
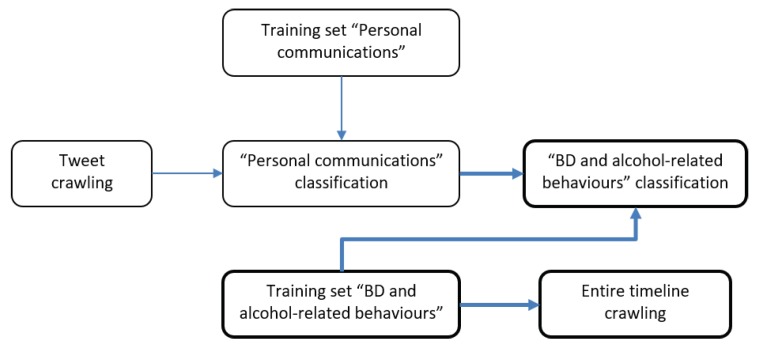
Classification flow using dataset D4 (in bold Phase 3).

During the classification process we considered, at first, only the tweets of the 180 users (around 360 tweets) manually labelled as genuine in Phase 2. Of these tweets, only those reporting a potential BD behaviour were selected. By following this approach, only 45 of the 180 genuine users were considered as potentially at risk of BD. With respect to Phase 2, in Phase 3 only an RF classifier via 5-fold cross validation was applied to the set of tweets belonging to these 45 users as training set. We opted for RF since it is effective in general, and especially for text categorization [53,54]. As illustrated in Section 2.2.1, on average, only two tweets were gathered with respect to each user during the focused crawling process based on hashtags detailed in Section 2.1. Therefore, it has been deemed necessary and useful to collect the tweet entire history of the 45 users manually labelled as potential binge drinkers. For this reason, a further crawler (focused on the 45 users) was developed, and a total of 86,204 tweets were retrieved, for an average of 1959 tweets per user (dataset D4). The complete classification pipeline is shown in Figure 2.

## 3. Results

**Figure 3:**
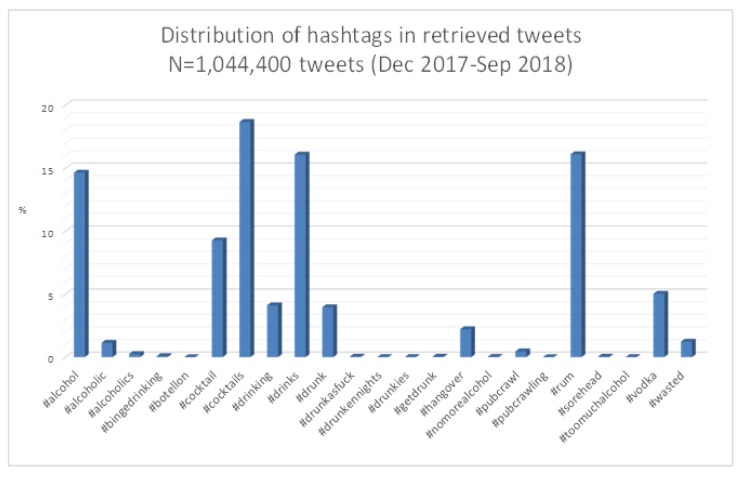
Distribution of hashtags used in the crawling process of binge drinking (BD)- and alcohol-related tweets.

In order to find tweets related to alcohol and BD (Phase 1 of the proposed approach), a preliminary textual content analysis was carried out to select those keywords (hashtags) supposed to be useful to identify alcohol-related tweets according to experts’ panel, as introduced in Section 2.1. Figure 3 shows that most frequent hashtags were #alcohol, #cocktail, #cocktails, #drinks, and #rum, followed by #drunk, #vodka, #drinking, and #hangover (see also Table A1 in Appendix A.1).

### 3.1. Dataset Characteristics

We extracted three seasonal waves of tweets: 409,788 from December 2017 to March 2018; 316,541 from April to June 2018; and 318,071 from July to September 2018. Table 1 summarizes the characteristics of both users and tweets from the three datasets. The average number of daily tweets was similar across time-periods, though the number of tweets was larger in winter (December 2017–March 2018). The majority of tweets was not in a favourite list in all time-periods (December–March 74%; April–June 88%; July–September 89%) or was liked only once (December–March 13%; April–June 8%; July–September 7%). In addition, users’ followers, favourites and friends’ distributions were highly skewed, with the right tail of the distribution longer than the left one, meaning the existence of users with a very large number of followers, favourites and friends.

### 3.2. Identification of Real Users with Respect to Bots, Media, and Business Accounts

Since a source of noise was likely to mask personal communications among genuine users’, in Phase 2 we used a structured approach to distinguish tweets of likely genuine (non-retailers) users with respect to bots, media, and business accounts. Various features (e.g., number of tweets of a single account, average number of hashtags, mentions, and occurrences of personal pronouns per tweet) were extracted to distinguish real users from non-real ones. Along with these features, distinct words were manually identified as keywords suggesting a media- or retail-related content, and included as additional linguistic features. This facilitated the assignment of tweets containing similar “bad tokens” to the class of those produced by non-real users (Table 2).

**Figure 4:**
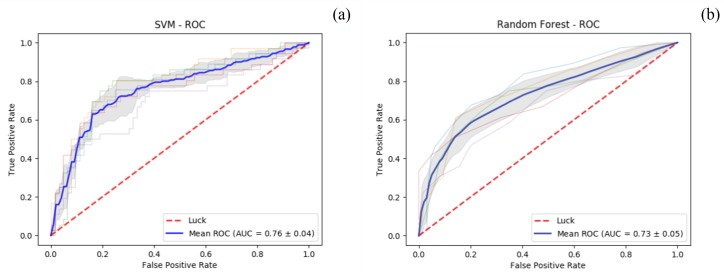
Receiver Operating Characteristic (ROC) curves and the Area Under the resulting ROC Curve (AUC) values for (**a**) Support Vector Machine (SVM) and (**b**) Random Forest (RF) classifiers.

The random subsample from the original set of tweets labelled by experts appeared to include a proportion of personal communications of about 36%. The classifier was trained on the subset labelled by experts, based on both SVM and RF models, and it showed a moderate fit in terms of performance in distinguishing personal communications (AUC values 0.76±0.04 and 0.73±0.05) when applied to the test set. When performing automatic classification on unlabelled data (in datasets D1, D2, and D3), the proposed approach estimated cumulative 45% of personal communications. The ROC curves and AUC average values for the SVM and RF classifiers are shown in Figure 4a,b.

#### Details on Evaluation Metrics

A ROC curve shows the performance of a classification model at all classification thresholds. In a classification task, a score s(i,c) is predicted for each item *i*, where the score denotes the probability that the item belongs to a class *c*. Therefore, it is possible to test different values for a threshold *t*, such that, in binary classification, s(i,c)≥t is interpreted as predicting *c* (i.e., the positive class), and s(i,c)<t is interpreted as predicting $\bar{c}$ (i.e., the negative class). The positive class is represented by genuine users, while the negative one by retail users. The ROC curve plots two parameters: (i) The True Positive Rate (TPR) and (ii) the False Positive Rate (FPR). The TPR is defined as $\frac{TP}{TP+FN}$, while the FPR as $\frac{FP}{FP+TN}$, where TP, FP, TN, and FN stand for True Positives: The number of items correctly classified (positive class); False Positives: The number of items incorrectly classified as belonging to the positive class; True Negatives: The number of items correctly classified (negative class); False Negatives: The number of items incorrectly classified as belonging to the negative class. AUC provides an aggregate measure of performance across all possible classification threshold values (ranging from 0 for model predictions 100% wrong to 1 for predictions 100% correct).

**Figure A1:**
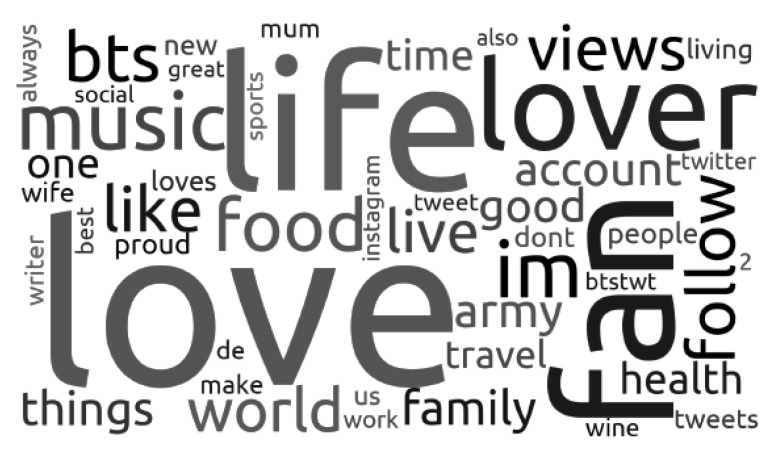
Most Frequently Reported Unigrams, Excluding Stop-Words: December 2017–March 2018.

**Figure A3:**
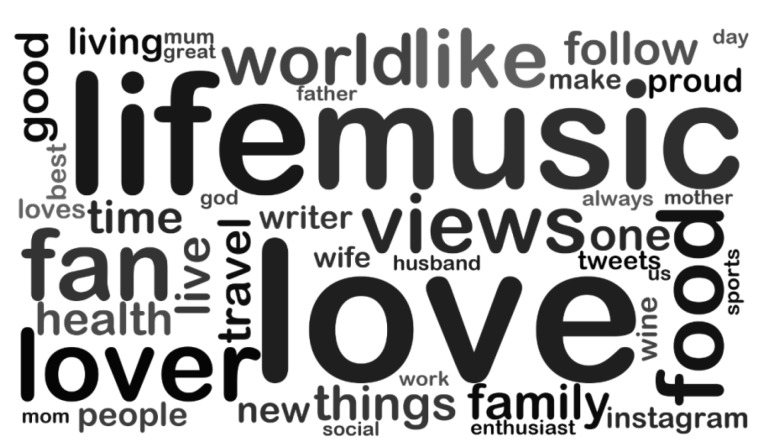
Most frequently reported unigrams, excluding stop-words: July 2018–September 2018.

A portion of bot, media and business users was identified as genuine users, i.e., some false positives were produced. Therefore, to get an in-depth focus on these classification results, we performed a simple content analysis to investigate occurrences and patterns of words within the tweets associated with users identified as genuine in D1, D2, and D3 by the automatic classification process. The text of these tweets was divided into sequences of *n* contiguous words occurring within a single tweet (*n*-grams). For each dataset (i.e., D1, D2, and D3), the resulting list was made up of the *n*-grams most frequently used by users identified as genuine. Table 3 reports the bigrams and trigrams more frequently used in each time-period, while Figure A1–Figure A3 in Appendix A.2 show the word clouds of the most relevant unigrams mentioned by the automatically identified genuine users. Bigrams and trigrams were more informative as compared with unigrams, since they provided some contextual information. As it emerges from Table 3, some retained bigrams, such as “mental health”, “public health”, and trigrams, such as “need help tweet”, might likely belong to profiles of users who help people and deal with public health issues (e.g., doctors, experts, journalists) and do not strictly represent personal communications. Furthermore, trigrams showed that some profiles, posting tweets about alcohol (e.g., bartenders), often include in their tweets an explicit indication to ban the consumption of alcohol by minors according to specific minimum legal age, e.g., “(don’t) share anyone 21”, “must (be) 21 (to) follow”, and “please drink responsibly”.

### 3.3. Identification of Potential Binge Drinkers

Table 4 shows the characteristics of the subsample constituted by the 45 users manually labelled as potential binge drinkers. For each user, the entire tweet history was considered, including on average 1959 tweets per user. Similarly to the whole sample, the majority of tweets was not in a favourite list or was liked only once. Statuses, followers and friends counts were consistent with the entire sample characteristics. However, a less skewed distribution was observed, since only personal communications were likely to be included (no media, bot, or business accounts). On average, these users have been registered in Twitter for a longer period of time. Since we were able to distinguish genuine users from commercial accounts with a pretty satisfactory accuracy (Phase 2, Section 3.2), we tried to further automatically identify those users who were likely to binge drink, by implementing an RF classifier trained on the set of 45 users labelled as binge drinkers and their linguistic features. The training of the RF classifier was carried out by considering both: (i) The original training dataset made by around two tweets per user (90 tweets); and (ii) the training dataset constituted by the entire tweet history of the 45 users (on average, 1959 tweets per user). The proposed approach to automatically classify binge drinkers did not reach satisfactory results with both strategies, though in the latter, given the greater number of tweets per user, accuracy improved (AUC values 0.67±0.05).

**Figure 5:**
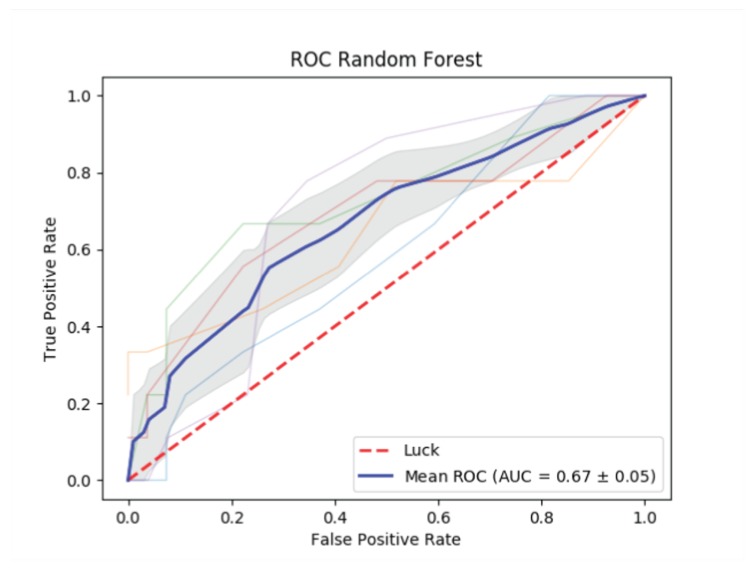
BD and alcohol-related behaviours characterization: ROC curve and AUC value based on a random forest classifier, for tweets in the index period + user’s entire timeline.

The ROC curve and the AUC value obtained by the RF classifier for the second training dataset are shown in Figure 5.

Finally, similarly to users identified as genuine in datasets D1, D2, and D3, we performed a basic content analysis to investigate occurrences and patterns of words within the whole sample of tweets from the 45 users manually identified as potential binge drinkers. Bigrams and trigrams more frequently used by these subjects (dataset D4) are reported in Appendix A.3. They did not appear to recur across different tweets. However, *n*-grams in dataset D4 were more likely to be related to personal matters as compared with those in datasets D1–D3, including also some suggestions of risky behaviors (e.g., “currently drunk abandon”; “drunk abandon building”).

**Figure A4:**
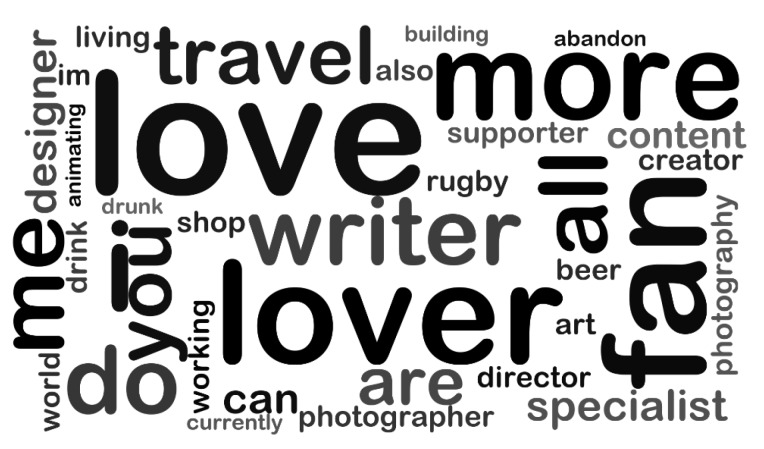
Most frequently reported unigrams, excluding stop-words, for the 45 potential binge drinkers (dataset D4).

Figure A4 in Appendix A.4 shows the wordcloud of the most relevant unigrams for the 45 binge drinkers in dataset D4, with unigram dimension proportional to frequency.

## 4. Discussion

In the current exploratory study, we developed a systematic process that enabled an analysis of Twitter User-Generated Content in terms of alcohol-related behaviours aimed at identifying language and shared topics of potential binge drinkers. We aimed to gain insight into specific topics and patterns helpful to envisage preventive approaches to recognize and target those people who are at risk of BD. The study assessed a technique to automatically identify potential binge drinkers, by testing a classifier on the contents of tweets. Since alcohol-related tweets were frequently associated with media and/or business activities, personal communications were automatically distinguished from this “noise” from data, by involving a team of experts that manually identified potential noise indicators (e.g., bad tokens). These bad tokens and other features connected to users and their contents were employed to classify with a reasonable accuracy genuine users with respect to “retail” ones.

Considering genuine users, when assessing the performance of the supervised machine learning classifier for automatically identifying potential binge drinkers, we obtained not completely satisfactory results in terms of accuracy. This is consistent with previous evidence showing similar models exploring mental health disorders from Twitter are often fuzzy and unstable [43]. Moreover, in our study, manual coding seems a crucial step in order to perform analyses based on machine learning classifiers, since it allows to train the algorithm model according to tweet’s characteristics vector and linguistic features in order to identify the target group (i.e., people at risk of BD and alcohol-related behaviours). Potential explanations of this difficulty include the need to consider new and changeable alcohol and drug use practices and related slang terminology, in order to identifying contents unequivocally related to BD. Manually labelled datasets to train algorithms able to identify alcohol-related contents have to deal with ambiguities in tweets when carrying out manual coding and need appropriate metrics to assess inter-coder reliability [55]. Furthermore, features selection process should be improved including all suitable *n*-grams that could be considered as bad tokens to let existing classification algorithms working more efficiently. Alternatives may involve patterns of use of Twitter, since people who are likely to BD are very much similar to standard users in terms of statuses, number of followers, friends and likes. Users identified as at risk convey a small proportion of tweets on BD, as compared with their entire bulk of tweets. People at risk are likely to share vocabulary and language, despite “background noise” from Twitter: We certainly do not expect a user to continually post messages regarding his/her problematic behaviour. In addition, slang expressions and number restrictions of the maximum characters of a tweet have a strong effect on tweets writing style, making the analysis complex. Magnitude and relevance of specific features might be exploited from n-grams and relevant analyses. Despite the actual identification of unique people with BD from their tweets is unfeasible, and probably not appropriate from an ethics perspective, these features might inform targeted preventive programs and focused campaigns, possibly benefiting from the cooperation of social media like Twitter that users clearly choose to express their BD characteristics.

The epidemiological approach to Twitter data represents an important challenge, since extrapolating knowledge from big data, including Twitter streams, and managing different textual contents possibly require more advanced computational methods to mine user profiles descriptions. These would allow to handle further metadata to take into account relevant individuals’ demographic characteristics in the analysis [56,57]. Surveys from social platforms are thus hardly comparable with standard epidemiological studies, rather they bring additional limitations. Privacy concerns emerge at different levels. According to a recent study [58], Twitter users appear to be unfamiliar with Twitter warning about the platform opportunity to broadly and instantly disseminate information or content like photos, videos, and links to a wide range of users, customers, services, and organizations, including researchers and public health agencies [59]. Thus, focusing on users’ perceptions about research on Twitter and how contextual factors are perceived, some best practices were identified. These include anonymizing identifying information when quoting tweets, not quoting tweets verbatim, honoring Twitter users’ efforts to control their personal data by omitting private and deleted information, using larger datasets [58,60]. Furthermore, users feel more comfortable with the idea of tweets being analyzed by a computer rather than read by humans. Thus, the development of automated tools might contribute to ethical practices and research implications, though outside the standard framework of research ethics. Algorithms should pursue the maximum benefit minimising the risk of potential harm during data collection, analysis and publication, while researchers should assess algorithms’ performance and routinely test them for effectiveness, avoiding the mislabelling of content [61]. Furthermore, discarding re-tweets may be considered a discretionary choice, since we aimed at preliminarily investigating individual-level data on social networking about alcohol-related behaviours. Multi-level data about re-tweet contents that users think will resonate with their followers is matter for future research.

We acknowledge the discretionary nature involving both the selection of hashtags and the supervised learning procedure chosen, as well as the linguistic feature analysis run. Moreover, we cannot assume that people who tweet on alcohol-related behaviours do actually use alcohol, though it is a likely linguistic proxy measure [62]. Moreover, we considered a single platform, i.e., Twitter, though how BD specific characteristics would match with certain features (public vs. private) of different messaging apps, e.g., WhatsApp or Line, remains to be explored. Finally, Twitter streams were sampled multiple times to reduce the impact of Twitter restrictions on the amount of data that can be collected through Twitter public APIs.

## 5. Preventive Implications and Conclusions

Behavioral and universal prevention programs have shown limited evidence in reducing BD, though its impact remains a cause for concern. Emerging issues such as BD may benefit from Twitter research focusing on behaviours less likely to be addressed in epidemiological research. Based on surveillance-like data from Twitter, strategies may be implemented encouraging awareness of the negative consequences of hazardous drinking, delivering a preventive message about BD. Likelihood of targeted behaviour patterns and the identification of target groups or places at high risk for unhealthy behaviours may represent key, high-resolution information to inform relevant stakeholders responsible for preventive policies [63]. Specifically, detecting real users reporting BD and alcohol-related risky behaviours on social media appears as a complex but promising approach deserving a deeper investigation in future studies.

## Figures and Tables

**Table 1:** Characteristics of users and tweets.

	**D1**	**D2**	**D3**
*Time period*	December 2017–March 2018	April–June 2018	July–September 2018
*Number of days*	96	77	80
*Number of tweets*	409,788	316,541	318,071
*Tweet favorite count*			
0	73.94%	88.42%	89.23%
1–5	23.62%	11.15%	10.36%
>5	2.45%	0.43%	0.41%
*Retweeted at least once*	45.85%	54.49%	50.11%
*Unique users*	144,614	129,808	131,161
Users’ characteristics *			
*Years since account*			
*creation median* (*iqr*)	5 (3–8)	6 (3–8)	6 (3–9)
*Statuses count median* (*iqr*)	2482 (554–9751)	3039 (663–12,376)	2872 (657–11,523)
*Average number of tweets*			
*per user in the time-period*	2.78	2.22	2.35
*Followers count median* (*iqr*)	353 (88–1317)	416 (104–1531)	411 (114–1449)
*Favorites count median* (*iqr*)	756 (116–3598)	1071 (171–5295)	990 (155–4849)
*Friends count median* (*iqr*)	477 (160–1361)	516 (171–1500)	509 (177–1438)
*URL in user’s profile*	52.59%	51.11%	53.36%

* Using last observation per user in the selected time-period in case of multiple information. *iqr* = interquartile range.

**Table 2:** Sets of bad tokens extracted from the text of the tweet, the user description and the user nickname.

Tweet Text	User Description	User Nickname
abuse	addiction	addiction
ad	advertising	bar
addict/addiction	bar	blog
bar	blog	book
book	boutique	bot
discount	business	business
disease	charity	country
dutyfree	commercial	disease
free	corporate	distillery
freetickets	crowdfunding	drink
gift	dependence	fitness
hotline	discounts	food
illness	distillery	game
magazine	editor	grocery
marketing	events	hotel
masterclass	fitness	journal
motivation	follow	lifestyle
official	free	magazine
page	game	marketing
quit/quitting	gifts	meal
recipe	gin	natural
recovery	help	news
shipped	inquires	official
shop	magazine	performance
sobriety	marketing	recipe
sponsor/sponsored	official	recovery
stop	organisation	renascence
treatment	page	shop
t-shirt	promotional	social
tutorial	prosecco	spotlight
win	recipes	town
	recovery	travel
	reservations	tweet
	shipped	win
	shop	
	store	
	travel	
	treatment	
	wodka	

**Table 3:** Most frequently reported *n*-grams in the three time periods.

n=2	Frequency (Occurrence) >55			n=3	Frequency (Occurrence)		
	**D1**	**D2**	**D3**		**D1**	**D2**	**D3**
fan account	-	254	63	must 21 follow	124	36	44
social media	238	173	197	behalf diageo brands	99	23	28
love life	216	155	172	share anyone 21	96	27	32
family friends	193	139	153	working behalf diageo	95	28	34
animal lover	184	153	145	21 follow please	95	25	33
love family	159	133	139	diageo brands must	84	19	23
live life	142	182	143	brands must 21	84	18	23
music lover	141	104	144	follow please enjoy	57	-	16
husband father	139	135	174	season ticket holder	55	57	51
mum 2	138	79	74	please drink responsibly	54	22	21
21 follow	137	-	-	please enjoy responsibly	54	-	18
must 21	134	-	-	responsibly share anyone	47	14	18
living life	130	101	125	drink responsibly share	39	14	-
good food	130	90	97	love family friends	39	27	29
wife mother	123	117	135	bts fan account	-	27	-
loving life	117	80	98	fan account btstwt	-	26	-
mental health	116	160	150	live life smiling	-	25	-
share anyone	115	-	-	site last tweet	-	20	-
mum 3	115	89	72	god family country	-	18	21
craft beer	111	85	114	link last tweet	-	18	-
follow us	110	-	81	love love peace	-	18	-
food wine	103	80	91	url last tweet	-	16	-
public health	102	162	161	good food good	-	15	-
follow please	101	-	-	love good food	-	15	-
part time	100	84	68	one day time	-	29	38
follow back	99	123	119	live laugh love	-	18	31
anyone 21	99	-	-	work hard play	-	33	25
love music	99	97	102	live love laugh	-	22	25
food drink	96	85	85	hard play hard	-	21	-
working behalf	95	-	-	official twitter account	-	33	24
dog lover	93	64	96	love life live	-	17	23
video games	89	77	85	live life full	-	25	22
happily married	89	81	74	live life fullest	-	22	22
life love	89	73	83	makes dream work	-	22	21
love travel	86	74	60	crazy cat lady	-	25	19
official twitter	86	76	66	die hard fan	-	18	19
sports fan	83	95	77	love life love	-	15	19
mum two	83	63	65	life one day	-	-	18
views expressed	77	87	77	living life fullest	-	-	18
twitter account	-	76	-	wife mother grandmother	-	-	18
god family	-	-	72	follow account geotargeted	-	24	18
lover things	-	70	83	help tweet us	-	24	18
human rights	-	67	-	need help tweet	-	24	18
last tweet	-	65	-	tweet us careerarc	-	24	18
season ticket	-	60	-	follow follow back	-	-	17
life short	-	59	-	follow us instagram	-	-	17

**Table 4:** Characteristics of users labelled as potential binge drinkers.

**Characteristics**	**N or %**
*Unique users*	45
*Number of tweets* (*users’ entire history*)	86,204
*Average number of tweets per user*	1959
*Tweet favorite count*	
0	83.16%
1–5	15.95%
>5	0.90%
Users’ characteristics *	**Median (** ***iqr*** **)**
*Years since account creation*	8 (7–10)
*Statuses count*	2664 (849–9602)
*Followers count*	238 (86–525)
*Favorites count*	1085 (111–3075)
*Friends count*	460 (166–715)
*URL in user’s profile* (%)	45.45%

* Using last observation per user in the selected time-period in case of multiple information. *iqr* = interquartile range.

**Figure A2:**
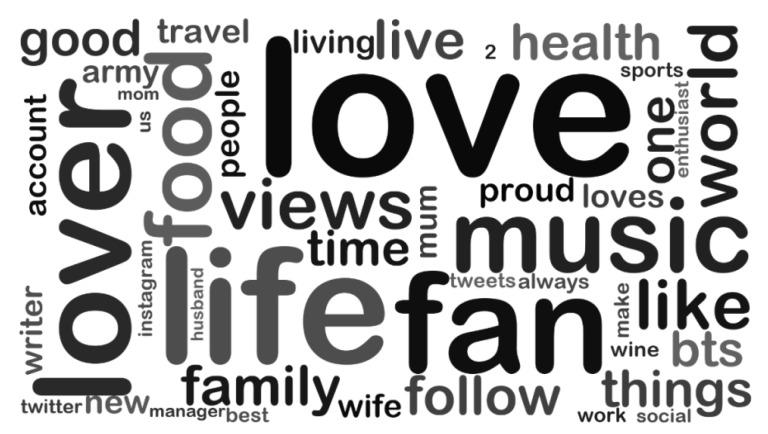
Most frequently reported unigrams, excluding stop-words: April 2018–June 2018.

## Appendix A. Hashtags Distribution and Most Frequently Reported n-Grams

### Appendix A.1. Hashtags Distribution across Datasets D1, D2, and D3

**Table A1:** Hashtags used to crawl alcohol-related tweets in three different time-periods.

	D1	D2	D3	
Hashtags	December 2017–March 2018	April–June 2018	July–September 2018	Total Sample
*N* (%)	N= 409,788	N= 316,541	N= 318,071	N= 1,044,400
**#alcohol**	**59,387 (14.49)**	**47,658 (15.06)**	**45,777 (14.39)**	**152,822 (14.63)**
#alcoholic	4061 (0.99)	4231 (1.34)	3594 (1.13)	11,886 (1.14)
#alcoholics	1116 (0.27)	711 (0.22)	756 (0.24)	2583 (0.25)
#bingedrinking	313 (0.08)	308 (0.10)	249 (0.08)	870 (0.08)
#botellon	3 (0.001)	4 (0.001)	3 (0.001)	10 (0)
**#cocktail**	**34,431 (8.40)**	**31,901 (10.08)**	**30,391 (9.55)**	**96,723 (9.26)**
**#cocktails**	**61,505 (15.01)**	**66,746 (21.09)**	**66,618 (20.94)**	**194,869 (18.66)**
#drinking	14,745 (3.60)	14,160 (4.47)	14,035 (4.41)	42,940 (4.11)
**#drinks**	**50,264 (12.27)**	**56,222 (17.76)**	**61,320 (19.28)**	**167,806 (16.07)**
#drunk	16,516 (4.03)	12,509 (3.95)	12,330 (3.88)	41,355 (3.96)
#drunkasfuck	76 (0.02)	278 (0.09)	87 (0.03)	441 (0.04)
#drunkennights	35 (0.01)	18 (0.01)	30 (0.01)	83 (0.01)
#drunkies	36 (0.01)	29 (0.01)	46 (0.01)	111 (0.01)
#getdrunk	175 (0.04)	122 (0.04)	170 (0.05)	467 (0.04)
#hangover	9265 (2.26)	6962 (2.20)	6912 (2.17)	23,139 (2.22)
#nomorealcohol	146 (0.04)	31 (0.01)	27 (0.01)	204 (0.02)
#pubcrawl	1513 (0.37)	1737 (0.55)	1600 (0.50)	4850 (0.46)
#pubcrawling	7 (0.002)	9 (0.003)	5 (0.002)	21 (0)
**#rum**	**67,812 (16.55)**	**51,166 (16.16)**	**49,078 (15.43)**	**168,056 (16.09)**
#sorehead	185 (0.05)	119 (0.04)	123 (0.04)	427 (0.04)
#toomuchalcohol	63 (0.02)	35 (0.01)	55 (0.02)	153 (0.01)
**#vodka**	**15,624 (3.81)**	**18,876 (5.96)**	**18,014 (5.66)**	**52,514 (5.03)**
#wasted	3286 (0.80)	2709 (0.86)	6851 (2.15)	12,846 (1.23)

In bold hashtag occurrence > 5% in the total sample.

### Appendix A.3. Most Frequently Reported n-Grams for the 45 Users Identified as Binge Drinkers by Considering Dataset D4

#### Appendix A.3.1. Bigrams (Bigram, Cardinality)

[(’abandon building’, 1), (’currently drunk’, 1), (’drunk abandon’, 1), (’wmcretail leading’, 1), (’specialist animating’, 1), (’space operating’, 1), (’place making’, 1), (’ceo wmcretail’, 1), (’animating retail’, 1), (’operating markets’, 1), (’making consultants’, 1), (’retail space’, 1), (’leading specialist’, 1), (’consultants views’, 1), (’markets live’, 1), (’live events’, 1), (’events place’, 1), (’mother 4’, 1), (’4 fab’, 1), (’tv addict’, 1), (’lover say’, 1), (’40 something’, 1), (’addict cat’, 1), (’wife mother’, 1), (’sons tv’, 1), (’cat lover’, 1), (’fab sons’, 1), (’something wife’, 1), (’solo designer’, 1), (’designer educational’, 1), (’educational content’, 1), (’content brand’, 1), (’illustrator solo’, 1), (’brand communications’, 1), (’manutd thebigclash’, 1), (’kayoss sweetshot’, 1), (’sweetshot gettipsy’, 1), (’aidoniaaaaa favourite’, 1), (’though chip’, 1), (’favourite though’, 1), (’chip kayoss’, 1), (’thebigclash aidoniaaaaa’, 1), (’writer htcwrestling’, 1), (’guy aowrestlingca’, 1), (’father interviewervideo’, 1), (’interviewervideo guy’, 1), (’htc’, 1), (’friendly stitcher’, 1), (’htcwrestling people’, 1), (’aowrestlingca writer’, 1)]

#### Appendix A.3.2. Trigrams (Trigram, Cardinality)

[(’currently drunk abandon’, 1), (’drunk abandon building’, 1), (’events place making’, 1), (’live events place’, 1), (’markets live events’, 1), (’place making consultants’, 1), (’retail space operating’, 1), (’specialist animating retail’, 1), (’animating retail space’, 1), (’operating markets live’, 1), (’space operating markets’, 1), (’ceo wmcretail leading’, 1), (’leading specialist animating’, 1), (’wmcretail leading specialist’, 1), (’making consultants views’, 1), (’something wife mother’, 1), (’mother 4 fab’, 1), (’fab sons tv’, 1), (’tv addict cat’, 1), (’addict cat lover’, 1), (’sons tv addict’, 1), (’4 fab sons’, 1), (’40 something wife’, 1), (’cat lover say’, 1), (’wife mother 4’, 1), (’content brand communications’, 1), (’designer educational content’, 1), (’solo designer educational’, 1), (’educational content brand’, 1), (’illustrator solo designer’, 1), (’€ manutd €’, 1), (’though chip kayoss’, 1), (’chip kayoss sweetshot’, 1), (’€ thebigclash €’, 1), (’favourite though chip’, 1), (’manutd € thebigclash’, 1), (’aidoniaaaaa favourite though’, 1), (’kayoss sweetshot gettipsy’, 1), (’thebigclash € aidoniaaaaa’, 1), (’manutd thebigclash aidoniaaaaa’, 1), (’stitcher htc podcast’, 1), (’father interviewervideo guy’, 1), (’htcwrestling people friendly’, 1), (’husband father interviewervideo’, 1), (’aowrestlingca writer htcwrestling’, 1), (’friendly stitcher htc’, 1), (’writer htcwrestling people’, 1), (’interviewervideo guy aowrestlingca’, 1), (’guy aowrestlingcawriter’, 1), (’people friendly stitcher’, 1)]
